# Native CGRP Neuropeptide and Its Stable Analogue SAX, But Not CGRP Peptide Fragments, Inhibit Mucosal HIV-1 Transmission

**DOI:** 10.3389/fimmu.2021.785072

**Published:** 2021-12-08

**Authors:** Jammy Mariotton, Anette Sams, Emmanuel Cohen, Alexis Sennepin, Gabriel Siracusano, Francesca Sanvito, Lars Edvinsson, Nicolas Barry Delongchamps, Marc Zerbib, Lucia Lopalco, Morgane Bomsel, Yonatan Ganor

**Affiliations:** ^1^ Laboratory of Mucosal Entry of HIV-1 and Mucosal Immunity, Department of Infection, Immunity and Inflammation, Institut Cochin, Université de Paris, INSERM U1016, CNRS UMR8104, Paris, France; ^2^ Department of Clinical Experimental Research, Glostrup Research Institute, Copenhagen University Hospital, Copenhagen, Denmark; ^3^ Emerging Bacterial Pathogens Unit, IRCCS San Raffaele Hospital, Milan, Italy; ^4^ Pathology Unit, Division of Experimental Oncology, IRCCS San Raffaele Hospital, Milan, Italy; ^5^ Urology Service, GH Cochin-St Vincent de Paul, Paris, France; ^6^ Immunobiology of HIV, Division of Immunology, Transplantation and Infectious Diseases, San Raffaele Scientific Institute, Milan, Italy

**Keywords:** CGRP, HIV-1, humanized BLT mice, Langerhans cells, SAX, STAT4

## Abstract

**Background:**

The vasodilator neuropeptide calcitonin gene-related peptide (CGRP) plays both detrimental and protective roles in different pathologies. CGRP is also an essential component of the neuro-immune dialogue between nociceptors and mucosal immune cells. We previously discovered that CGRP is endowed with anti-viral activity and strongly inhibits human immunodeficiency virus type 1 (HIV-1) infection, by suppressing Langerhans cells (LCs)-mediated HIV-1 trans-infection *in-vitro* and mucosal HIV-1 transmission *ex-vivo*. This inhibition is mediated *via* activation of the CGRP receptor non-canonical NFκB/STAT4 signaling pathway that induces a variety of cooperative mechanisms. These include CGRP-mediated increase in the expression of the LC-specific pathogen recognition C-type lectin langerin and decrease in LC-T-cell conjugates formation. The clinical utility of CGRP and modalities of CGRP receptor activation, for inhibition of mucosal HIV-1 transmission, remain elusive.

**Methods:**

We tested the capacity of CGRP to inhibit HIV-1 infection *in-vivo* in humanized mice. We further compared the anti-HIV-1 activities of full-length native CGRP, its metabolically stable analogue SAX, and several CGRP peptide fragments containing its binding C-terminal and activating N-terminal regions. These agonists were evaluated for their capacity to inhibit LCs-mediated HIV-1 trans-infection *in-vitro* and mucosal HIV-1 transmission in human mucosal tissues *ex-vivo*.

**Results:**

A single CGRP intravaginal topical treatment of humanized mice, followed by HIV-1 vaginal challenge, transiently restricts the increase in HIV-1 plasma viral loads but maintains long-lasting higher CD4+ T-cell counts. Similarly to CGRP, SAX inhibits LCs-mediated HIV-1 trans-infection *in-vitro*, but with lower potency. This inhibition is mediated *via* CGRP receptor activation, leading to increased expression of both langerin and STAT4 in LCs. In contrast, several N-terminal and N+C-terminal bivalent CGRP peptide fragments fail to increase langerin and STAT4, and accordingly lack anti-HIV-1 activities. Finally, like CGRP, treatment of human inner foreskin tissue explants with SAX, followed by polarized inoculation with cell-associated HIV-1, completely blocks formation of LC-T-cell conjugates and HIV-1 infection of T-cells.

**Conclusion:**

Our results show that CGRP receptor activation by full-length CGRP or SAX is required for efficient inhibition of LCs-mediated mucosal HIV-1 transmission. These findings suggest that formulations containing CGRP, SAX and/or their optimized agonists/analogues could be harnessed for HIV-1 prevention.

## Introduction

CGRP is a 37 amino acid potent vasodilator neuropeptide secreted from peripheral sensory nerves, such as pain nociceptors, which plays important physiological and pathophysiological roles (1). The CGRP receptor is a heteromeric complex, composed of calcitonin receptor-like receptor (CLR), the transmembrane receptor activity-modifying protein 1 (RAMP1), and the intracellular receptor component protein (RCP) that is important for signaling (2). CGRP receptor antagonism has been proven effective against migraine, in which CGRP is detrimental, and several CGRP receptor antagonists and neutralizing antibodies (Abs) are used clinically (3, 4).

However, CGRP-mediated vasodilation is potentially protective, at least during hypertension and cardiovascular complications (5). Indeed, both CGRP and its long-acting metabolically stable analogue SAX (serinyl-CGRP_2-37_-amide with an albumin binding fatty acid moiety in the N-terminus) (6, 7), exert protective vascular pharmacological effects *in-vitro* (7) and *in-vivo* (8). Compared to CGRP, SAX has a longer half-life (6), but decreased potency (6, 7).

CGRP also directly modulates immune function in a vasodilator-independent manner, as part of the neuro-immune dialogue between CGRP-secreting mucosa-innervating nociceptors and resident mucosal immune cells (9). For instance, nociceptors associate with LCs and CGRP shifts LCs-mediated antigen presentation and cytokine secretion from Th1 to Th2/Th17 (10).

We previously reported that LCs are the early cellular targets of HIV-1 upon its mucosal entry in the inner foreskin, and subsequently transfer infectious virus to CD4+ T-cells (11, 12) in a process termed trans-infection. We further discovered that LCs express the components of the CGRP receptor (i.e., CLR, RAMP1 and RCP) (13, 14), and that CGRP modulates a multitude of cellular processes in LCs, which cooperate together to significantly inhibit LCs-mediated HIV-1 trans-infection *in-vitro* and mucosal HIV-1 transmission *ex-vivo* (13–15). Accordingly, CGRP increases expression of the LC-specific pathogen recognition C-type lectin langerin, and facilitates efficient viral degradation by diverting HIV-1 from endo-lysosomes towards faster viral proteasomal degradation. CGRP also decreases LCs surface expression of several adhesion molecules, leading to reduced conjugate formation with CD4+ T-cells. Importantly, although CGRP activates the canonical CGRP receptor cAMP/PKA signaling pathway in LCs (16), we found that the anti-HIV-1 effects of CGRP in LCs are mediated *via* non-canonical NFκB/STAT4 signaling, as pharmacological inhibitors of both NFκB (13) and STAT4 (14) completely abrogate CGRP-induced inhibition of HIV-1 trans-infection. Based on these observations, we suggested that CGRP agonists/analogues might be useful for prevention of mucosal HIV-1 transmission.

The N-terminus (residues 1–7, containing a disulfide bond between the cysteines at positions 2 and 7) and amidated C-terminus (residues 27–37) of CGRP interact independently with the CGRP receptor in a two-domain model, whereby the C-terminus first binds the receptor, facilitating subsequent binding and activation by the N-terminus (17). The N-terminal disulfide loop is crucial for agonistic activity, as the peptide fragment CGRP_8–37_ is an antagonist, and as several N-terminal peptide fragments of CGRP are low-potency agonists with anti-hypertensive function (18). Other CGRP peptide fragments, containing constrained N-terminus (i.e., truncated loop with only three residues) and/or introduced disulfide bridge in the C-terminus, yield analogues with affinities comparable to native CGRP (19).

Here we evaluated the inhibitory activity of CGRP in pre-clinical experiments, using a mucosal model of HIV-1 infection in humanized mice. We further determined the requirements of CGRP receptor activation for inhibition of HIV-1 trans-infection *in-vitro*, by comparing the anti-HIV-1 activities of full-length native CGRP, its analogue SAX, and several CGRP N-terminal fragments and N+C-terminal bivalent fragments. Finally, we compared CGRP and SAX for their capacity to inhibit mucosal HIV-1 transmission in human mucosal tissues *ex-vivo*.

## Materials and Methods

### Agonists and Antagonists

We used the following molecules: CGRP (1 mM stock solution in water; Sigma), biotinylated CGRP (1 mM stock solution in water; AnaSpec), SAX (1 mM stock solution in DMSO) prepared as we described (7), custom synthesized CGRP peptide fragments (1 mM or 10 mM stock solutions in water, for N-terminal or N+C-terminal fragments, respectively; United Biosystems), and the CGRP receptor antagonist BIBN4096 (10 mM stock solution in DMSO; Sigma).

### Cells and Tissues

Peripheral blood mononuclear cells (PBMCs) from healthy HIV-1 seronegative individuals were separated from whole blood by standard Ficoll gradient. CD4+ T-cells and CD14+ monocytes were purified from PBMCs using appropriate negative magnetic selection kits (Stemcell Technologies), according to the manufacturer’s instructions. Monocytes (10^6^ cells/well in 12-well plates) were differentiated into monocyte-derived LCs (MDLCs) in complete RPMI medium [RPMI 1640 medium, 10% fetal bovine serum, 2 mM glutamine, 100 U/ml penicillin, and 100 μg/ml streptomycin (Gibco Invitrogen)], supplemented with 100 ng/ml granulocyte-macrophage colony-stimulating factor (GM-CSF), 10 ng/ml interleukin 4 (IL4), and 10 ng/ml transforming growth factor beta 1 (TGFβ1) (R&D systems) as described (20), and used 7–9 days after differentiation.

Normal foreskin tissues were obtained from healthy adults undergoing circumcision (Urology Service, Cochin Hospital, Paris). Human penile tissues were obtained as part of our previous study (21).

### Virus and Infected Cells

Viral stocks of the HIV-1 molecular clones JRCSF and ADA, and the primary isolate 93BR029 (V29), both clade B with R5 tropism (NIH AIDS reagent program), were prepared by transfection of 293T cells or by amplification on phytohemagglutinin (PHA)/IL2-activated PBMCs, respectively, and quantified using the p24 Innotest HIV-1 ELISA (Fujirebio). HIV-1 V29-infected PBMCs were prepared as we reported (11).

### CGRP and HIV-1 Infection in Mice

CGRP (10 nM, 100 nM or 1 μM) was diluted in 30 μl sterile phosphate-buffered saline (PBS), alone or in combination with 1% hydrocortisone, and applied intravaginally for 6 h in normal female BALB/c mice (10 weeks old, 25–30 g, synchronized in estrous cycle). Spleen, lymph nodes, gut, liver, kidneys, and female reproductive system were then collected, and hematoxylin and eosin stained 3 μm paraffin sections were examined for histopathological analysis. Selected slides were stained after antigen retrieval with monoclonal Abs (Bio-Rd), including rat-anti-human CD3, rat-anti-mouse B220/CD45R (clone RA3-6B2), and rat-anti-mouse F4/80 (clone CI:A31), followed by rat on rodent horseradish peroxidase (HRP)-polymer, 3,3’-diaminobenzidine (DAB) as chromogen (Biocare Medical) and counterstaining with hematoxylin. Images were acquired with the AxioCam HRc using the AxioVision System SE64 (Zeiss).

Humanized female bone-marrow/liver/thymus (BLT) mice were prepared, inoculated and examined for HIV-1 infection at the Ragon Institute Human Immune System Mouse Program, according to their established protocols (see https://www.ragoninstitute.org/research/services/humanized-mouse/). Expression of human langerin was determined by immunohistochemistry of 4 μm vaginal tissue paraffin sections as we described (12), using goat-anti-human langerin Ab (R&D), the LSAB2-HRP System with DAB as substrate (Dako) and counterstaining with hematoxylin. For infection experiments, CGRP (500 nM or 5 μM) in 30 μl sterile PBS or PBS alone were topically applied onto the vaginal epithelium for 4 h, followed by topical vaginal challenge with 2 × 10^4^ TCID_50_ HIV-1 JRCSF.

### HIV-1 Trans-Infection and Langerin Expression

MDLCs (10^5^/well in 96 round-bottom well plates) were treated for 24 h at 37°C (200 μl/well final) with the indicated molar concentrations of CGRP, SAX or CGRP fragments. The CGRP receptor antagonist BIBN4096 was added 15 min before agonists. For langerin surface expression, MDLCs were washed and stained for 20 min on ice in a final volume of 50 µl PBS with a phycoerythrin (PE)-conjugated mouse monoclonal Ab against human langerin (clone DCGM4, Beckman Coulter), or matched isotype control. For HIV-1 trans-infection, MDLCs were washed and pulsed with HIV-1 ADA (1ng p24 corresponding to multiplicity of infection of 0.2) for 4 h. MDLCs were next incubated with autologous CD4+ T-cells or with green fluorescent protein (GFP)-reporter T-cells, and HIV-1 replication was measured in the co-culture supernatants using p24 ELISA (Fujirebio) or by evaluating GFP fluorescent by flow cytometry, as we described (13–15). Fluorescent profiles were acquired using a Guava easyCite and analyzed with the InCyte software (Merck-Millipore).

### STAT4 Western Blot (WB)

For PBMCs, cells (2 × 10^6^/sample) were activated with PHA (5 μg/ml) + IL2 (100 U/ml) for 48 h at 37°C, serum-starved overnight, and stimulated for 30 min at 37°C with either IL12 (10 ng/ml; R&D systems) or interferon alpha (IFNα, 5 × 10^4^ U/ml; pbl Assay Science). For MDLCs, cells (2 × 10^6^/sample) were re-suspended in complete RPMI medium without cytokines and rested overnight at 37°C. MDLCs were next treated for 24 h at 37°C with CGRP (0.1 μM), SAX (0.1 μM), CGRP_1–8_ (10 μM), or lipopolysaccharide (LPS, 10 μg/ml). The CGRP receptor antagonist BIBN4096 (1 μM) was added 15 min before agonists. The cells were then washed and stimulated for 30 min at 37°C with combination of IL12 + IFNα at the concentrations indicated above. PBMCs/MDLCs were subsequently lysed for 30 min on ice with 100 μl lysis buffer [50 mM Tris buffer pH = 7.5, 150 mM NaCl, 2 mM EDTA, 1% Triton X100, 0.1% SDS, 1:100 dilutions of phosphatase inhibitors II/III and protease inhibitor cocktail (Sigma)], followed by three cycles of 10 s vortex and 10 min incubation on ice. Lysates were centrifuged for 10 min at 4°C/13,200 rpm, and supernatants were collected and stored at −80°C. Protein contents in cell lysates were quantified using the BCA kit (Thermo Fisher) according to the manufacturer’s instructions, and 20 μg proteins were mixed with loading buffer (100 mM Tris pH 7.2, 5% β-mercaptoethanol, 12% glycerol, 5 mM EDTA, 5% SDS, 0.01% bromophenol blue), heated for 5 min at 95°C, run over a 10% SDS-PAGE, and transferred onto nitrocellulose membranes. Blocking was performed for 1 h at room temperature with blocking buffer (Tris-buffered saline (TBS), 0.5% Tween 20, and 0.5% dry milk). The blots were next incubated overnight at 4°C with commercial rabbit polyclonal Abs suitable for WB, directed against human STAT4 (Proteintech #13028-1AP, 0.5 μg/ml) or phosphorylated STAT4 (pSTAT4; R&D systems, #AF4319, 1 μg/ml), followed by 1:1,000 dilution of HRP-conjugated donkey-anti-rabbit IgG Ab (Southern Biotech) for 1 h at room temperature. Loading control was verified by incubation with goat polyclonal Ab to beta actin (abcam, 0.4 μg/ml), followed by 1:5,000 dilution of HRP-conjugated donkey-anti-goat IgG Ab (Promega). All Abs were diluted in blocking buffer, and pre-stained SDS-PAGE standard markers (ThermoFisher) were applied to determine molecular weights. Revelation was performed for 1–10 s with ECL-Prime chemiluminescence detection kit (Amersham). Images were acquired with the Fusion FX camera platform (Vilber Lournmat) and protein expression was quantified with ImageJ software (NIH).

### CGRP Entry and HIV-1 Transmission in Human Mucosal Tissue Explants

Polarized penile *fossa navicularis* tissue explants were prepared as we previously described (21), and exposed for 3 h to 500 nM or 5 μM biotinylated CGRP in 100 μl RPMI 1640 added to the apical side. After incubation, tissue penetration of biotinylated CGRP was examined by histochemistry of 4 μm paraffin sections as we described (21), using HRP-coupled streptavidin (Vector), followed by the red 3-amino-9-ethylcarbazole (AEC) HRP substrate (Dako) and counterstaining with hematoxylin. Images were acquired with an Olympus BX63F microscope using MetaMorph (Molecular Devices) and analyzed with ImageJ software.

For infection experiments, round (8 mm diameter) inner foreskin tissue pieces were placed in 24-well plates and incubated submerged for 24 h at 37°C with 1 ml complete RPMI medium, alone or supplemented with CGRP or SAX (1 μM, four explants per condition). The tissues were next washed, transferred to two-chamber transwell inserts (Sigma), and inoculated in a polarized manner for 4 h at 37°c with either non-infected or HIV-1 (V29)-infected PBMCs (in duplicates), as we described (11, 12).

Epidermal cell suspensions were prepared immediately after inoculation, using enzymatic digestion with dispase/trypsin as we described (11, 12). Pooled cells of each duplicate were resuspended in PBS, transferred to 96 round-bottom well plates and stained for 30 min on ice with 10 μl of fluorescein isothiocyanate (FITC)-conjugated mouse-anti-human CD1a, PE-conjugated mouse-anti-human CD8 and allophycocyanin (APC)-conjugated mouse-anti-human CD3 (BD Pharmingen) Abs, diluted in PBS in a final volume of 50 μl/well.

Dermal cell suspensions were prepared following washing of the explants, additional incubation for three days at 37°C submerged in 1 ml fresh medium, and subsequent enzymatic digestion with collagenase/DNase as we described (11, 12). Pooled cells of each duplicate were surface stained as above using FITC-conjugated mouse-anti-human CD3 Ab (Pharmingen), fixed, permeabilized, and stained for 30 min at room temperature with 1:160 dilution of PE-conjugated mouse-anti-human Ab to HIV-1 p24 and core antigens (Beckman Coulter). Fluorescent profiles were recorded using a Guava easyCyte and InCyte software.

### Data and Statistical Analysis

Data was analyzed using Prism software (GraphPad). Concentration-response curves were analyzed with the [log(agonist) vs. response (three parameters)] model for langrin upregulation and the [log(inhibitor) vs. normalized response − variable slope] model for HIV-1 trans-infection inhibition. The -log molar concentrations of agonists generating 50% response represented potencies (i.e., pEC_50_ and pIC_50_). Statistical significance was analyzed with the two-tailed Student’s t-test.

## Results

### CGRP Limits HIV-1 Infection *In-Vivo*

To test for the possible clinical utility of CGRP receptor agonism, we investigated the effects of CGRP in normal mice and in a mucosal model of HIV-1 infection in humanized mice.

**Figure 1:**
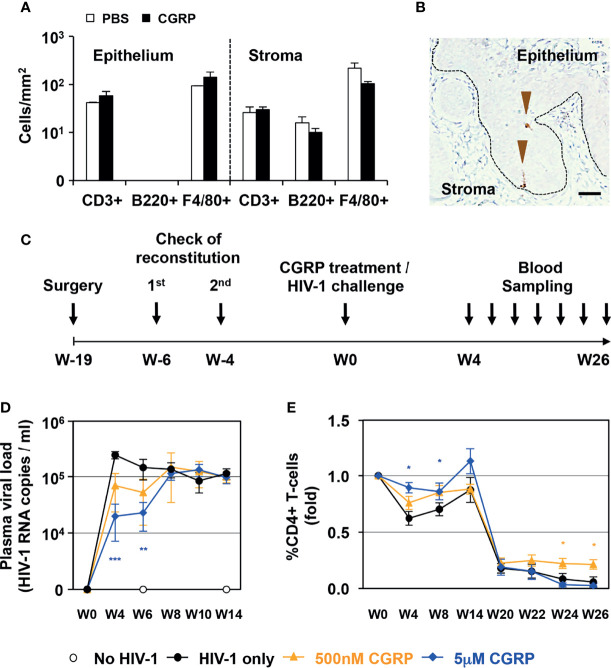
CGRP limits mucosal HIV-1 transmission *in-vivo*. **(A)** CGRP (1 μM) or PBS were topically applied intravaginally in normal female BALB/c mice (n = 3 animals per group). Vaginal tissue sections were examined by immunohistochemistry for the presence of CD3+ T-cells, B220+ B cells and F4/80+ macrophages. Shown are mean ± SEM cell densities expressed as cells/mm^2^ (of n = 3 independent experiments) in either the vaginal epithelium or stroma. **(B)** Representative image of the vaginal tissue of humanized BLT mice, showing expression of human langerin (arrowheads) in the epithelium; broken line denotes the basement membrane and scale bar = 20 μm. **(C)** Experimental schedule for preparing humanized female BLT mice, intravaginally applying CGRP or PBS followed by vaginal challenge with high-dose HIV-1 JRCSF, and subsequent blood sampling for measurement of plasma viral loads and CD4+ T-cells counts. **(D, E)** Shown are mean ± SEM (n = 5 BLT mice per group) of plasma viral loads (HIV-1 RNA copies/ml) or CD4+ T-cells percentages (fold). *p < 0.0500, **p < 0.0050, ***p < 0.0005; two-sided Student’s t-test.

First, as CGRP mediates vasodilator-dependent neurogenic inflammation that can result in immune cell recruitment, we topically applied CGRP onto the vagina of normal BALB/c mice for 6 h and examined potential toxicity and immune cells modulation. These experiments showed that CGRP, tested at 10 nM, 100 nM or 1μM, did not induce signs of toxicity and did not induce overt inflammation. Of note, in our routine experiments, treatment of MDLCs *in-vitro* with up to 10 μM CGRP for 24 h did not affect cell viability. At its highest concentration tested *in-vivo* of 1 μM, CGRP did not modify the distribution and/or density of T cells, B cells, and macrophages, neither in the epithelium nor in the stroma (**Figure 1A**).

Second, we used humanized BLT mice, which are suitable to study many aspects of HIV-1 infection, prevention and mucosal/vaginal transmission (22). We confirmed the reconstitution and presence of human langerin-expressing LCs within the vaginal epithelium (**Figure 1B**). We then topically applied CGRP onto the vagina of BLT mice for 4 h, followed by a vaginal challenge with high dose of cell-free HIV-1, as routinely used in this model (23). Such cell-free viral challenge permits to achieve productive infection, which would have been obtained using a much lower inoculum of cell-associated HIV-1 [i.e., that is transmitted more efficiently due to the formation of viral synapses between cell-associated HIV-1 and apical epithelial cells, leading to polarized budding of HIV-1, such as we reported in the inner foreskin (11, 12)]. Subsequently, we sampled blood at different time points for quantification of HIV-1 viral loads and CD4+ T-cell counts (**Figure 1C**).

These experiments showed that a single CGRP application dose dependently and significantly restricted the increase in plasma viral loads at two early time points (weeks 4 and 6, **Figure 1D**). CGRP treatment also significantly maintained higher CD4+ T-cells counts, both at the same early time points and also at the latest time points examined (weeks 24 and 26, **Figure 1E**).

These results show no signs of local CGRP-mediated toxicity *in-vivo.* In addition, CGRP exerts transient protection against the increase in HIV-1 viral loads, but long-lasting maintenance of higher CD4+ T-cell counts in HIV-1-infected BLT mice, providing proof-of-concept for the utility of CGRP *in-vivo*.

### CGRP and SAX, But Not CGRP Peptide Fragments, Inhibit HIV-1 Trans-Infection and Increase Langerin Surface Expression

**Figure 2:**
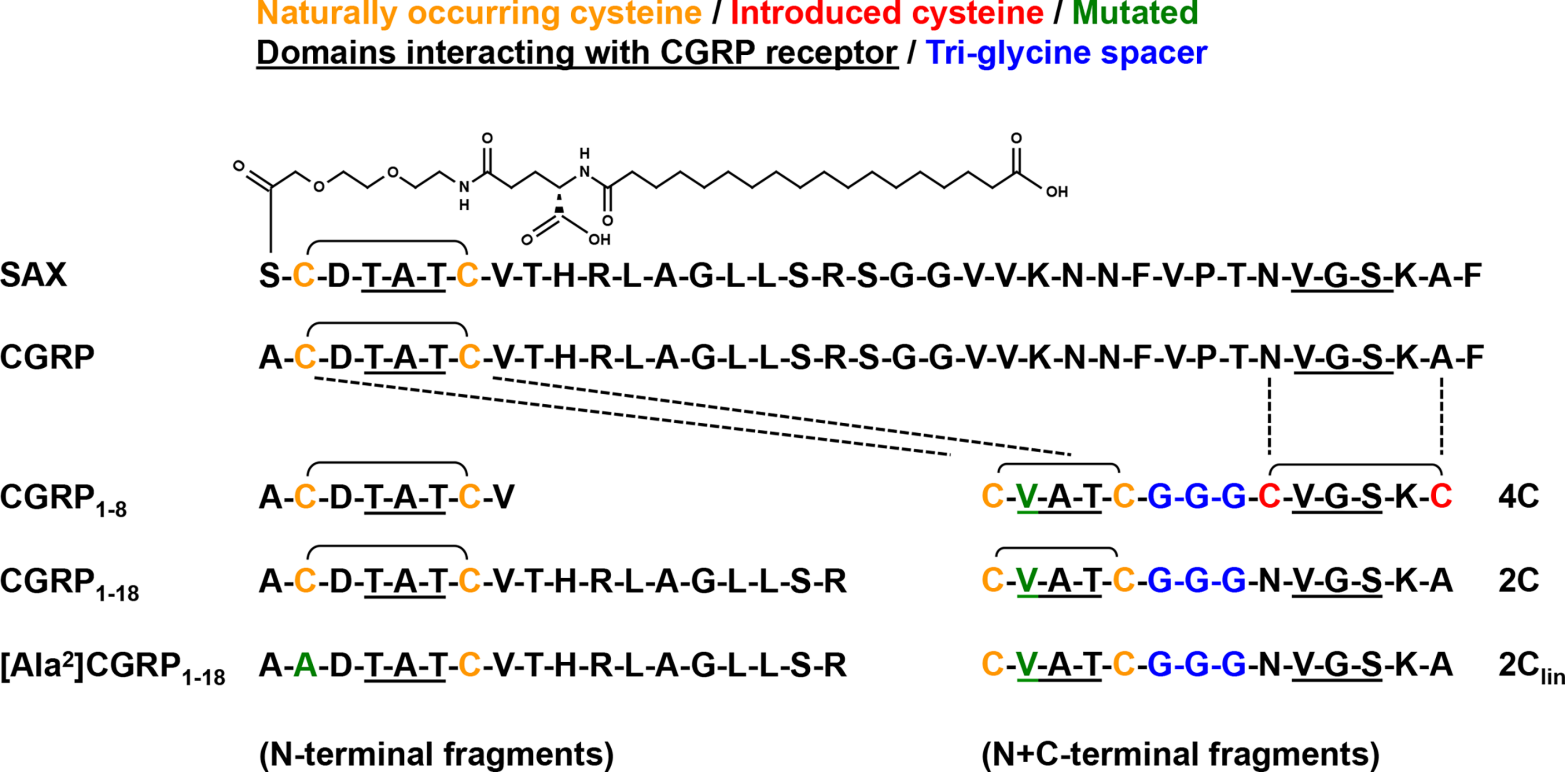
Sequences of CGRP, SAX and CGRP fragments. Amino acid sequences of the different agonists used in the current study, including full-length native CGRP; the long-acting metabolically stable CGRP analogue SAX; the N-terminal fragments CGRP_1–8_, CGRP_1–18_, and control mutated [Ala^2^]CGRP_1–18_; the bivalent N+C-terminal fragments 4C, 2C, and control 2C_lin_ lacking disulfide bonds.

To further determine the functional activities of CGRP receptor agonists, we compared the anti-HIV-1 inhibitory potential of CGRP, SAX, and several CGRP peptide fragments (**Figure 2**). As several CGRP N-terminal fragments are biologically active and exert anti-hypertensive functions (24), we tested the previously described CGRP_1–8_ (18) and CGRP_1–18_ (19) fragments, as well as the negative control mutated [Ala^2^]CGRP_1–18_ fragment (**Figure 2**, left). We also designed novel bivalent CGRP peptide fragments (**Figure 2**, right), by linking the previously described constrained CGRP N- and C-terminal regions (19), containing disulfide bonds either at both N/C-terminal regions or only at the N-terminal, with a tri-glycine spacer. These bivalent fragments were termed according to the number of their cystetin residues, namely 4C, 2C and the control 2C_lin_ devoid of disulfide bonds.

**Figure 3:**
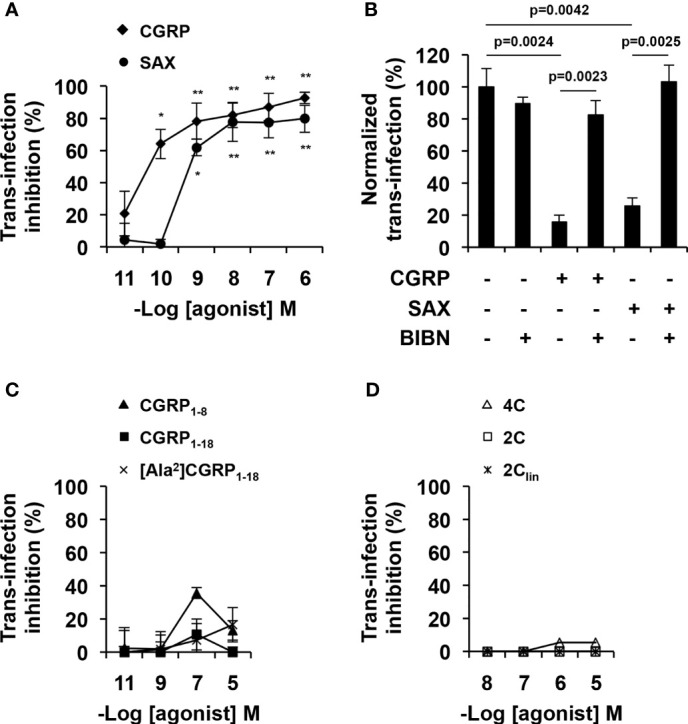
CGRP and SAX, but not CGRP peptide fragments, inhibit MDLCs-mediated HIV-1 trans-infection. MDLCs were left untreated or treated for 24 h with the indicated molar concentrations of CGRP, SAX or CGRP peptide fragments. In panel **(B)**, the CGRP receptor antagonist BIBN4096 (BIBN, 1 μM) was added 15 min before addition of agonists (0.1 μM). The cells were then pulsed with HIV-1 ADA for 4 h, washed, and incubated for 7 days with autologous CD4+ T-cells **(A–C)** or 3 days with HIV-1 GFP-reporter T-cells **(D)**. HIV-1 trans-infection and replication in T-cells was evaluated by p24 ELISA in the co-culture supernatants or by GFP expression and flow cytometry. Shown are mean- ± SEM percentages of HIV-1 trans-infection inhibition **(A, C, D)** or normalized **(B)**, derived from n = 5 **(A)** and n = 3 **(B–D)** independent experiments using MDLCs from different individuals. *p < 0.0500, **p < 0.0050, two-sided Student’s t-test.

MDLCs were treated for 24 h with CGRP, SAX or the different CGRP peptide fragments, pulsed with HIV-1, washed and co-cultured with autologous or GFP-reporter CD4+ T-cells. HIV-1 replication was next determined by measuring the content of the HIV-1 capsid protein p24 in the co-culture supernatant by ELISA or by evaluating GFP fluorescence using flow cytometry. In line with our previous results (13–15), CGRP strongly inhibited MDLCs-mediated HIV-1 trans-infection in a dose-dependent manner (**Figure 3A**). SAX also significantly inhibited HIV-1 trans-infection in a dose-dependent manner (**Figure 3A**), but had lower potency than CGRP, with pIC_50_ values [95% confidence intervals (CIs)] of 8.9 [9.9–7.9] compared to 10.2 [10.9–9.4], respectively. Of note, vehicle control for SAX treatment, i.e., 0.1% DMSO, had no significant effect. These inhibitory effects were mediated *via* activation of the CGRP receptor, as pre-incubation with the CGRP receptor antagonist BIBN4096 completely abrogated both CGRP- and SAX-mediated inhibition (**Figure 3B**). In contrast, none of the CGRP N-terminal (**Figure 3C**) and bivalent (**Figure 3D**) fragments significantly inhibited MDLCs-mediated HIV-1 trans-infection.

**Figure 4:**
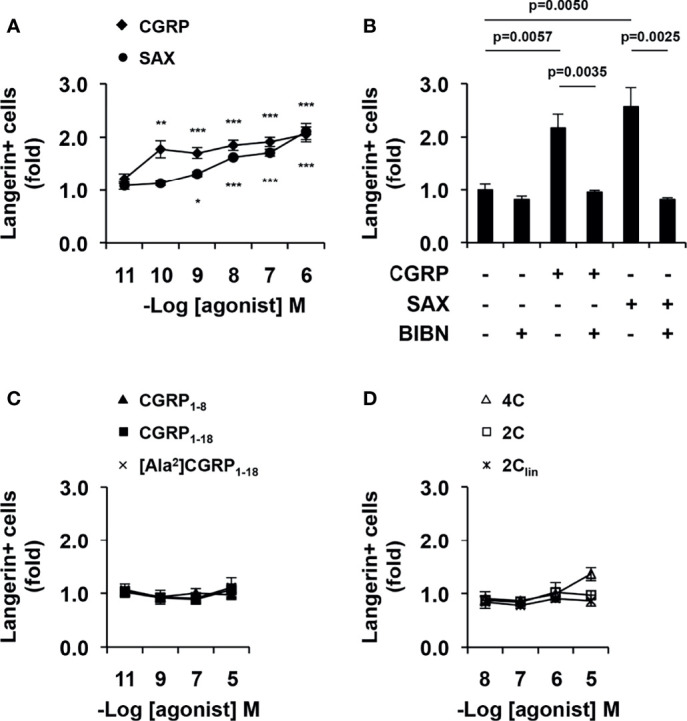
CGRP and SAX, but not CGRP peptide fragments, increase langerin expression in MDLCs. MDLCs were left untreated or treated for 24 h with the indicated molar concentrations of CGRP, SAX or CGRP peptide fragments. In panel **(B)**, the CGRP receptor antagonist BIBN4096 (BIBN, 1 μM) was added 15 min before addition of agonists (0.1 μM). The cells were then stained for surface langerin and examined by flow cytometry. Shown are mean ± SEM folds increase in the percentages of langerin+ cells, derived from n = 5 **(A, C)**, n = 4 **(B)** and n = 3 **(D)** independent experiments using MDLCs from different individuals. *p < 0.0500, **p < 0.0050, ***p < 0.0005, two-sided Student’s t-test.

We previously showed that one of the functional effects of CGRP during inhibition of HIV-1 trans-infection is related to upregulation of langerin surface expression in LCs (13–15). MDLCs were therefore treated with CGRP, SAX or CGRP peptide fragments for 24 h, and langerin surface expression was evaluated by flow cytometry. These experiments showed that both CGRP and SAX increased langerin expression in MDLCs in a dose-dependent manner (**Figure 4A**). As for inhibition of MDLC-mediated HIV-1 trans-infection, SAX had lower potency than CGRP, with pEC_50_ values of 8.3 [9.6–6.5] compared to 10.6 [11.5–9.7], respectively. Langerin upregulation was mediated *via* CGRP receptor activation, as the CGRP receptor antagonist BIBN4096 completely abrogated CGRP- and SAX-mediated increase in langerin expression (**Figure 4B**). In contrast, all CGRP fragments lacked agonistic activity and did not significantly increase langerin surface expression (**Figures 4C, D**).

These results show that CGRP and SAX inhibit HIV-1 trans-infection, which correlate with their ability to activate the CGRP receptor and increase langerin expression in MDLCs. In contrast, CGRP peptide fragments fail to increase langerin and inhibit HIV-1 trans-infection.

### CGRP and SAX, But Not CGRP_1–8_, Increase STAT4 Expression

**Figure 5:**
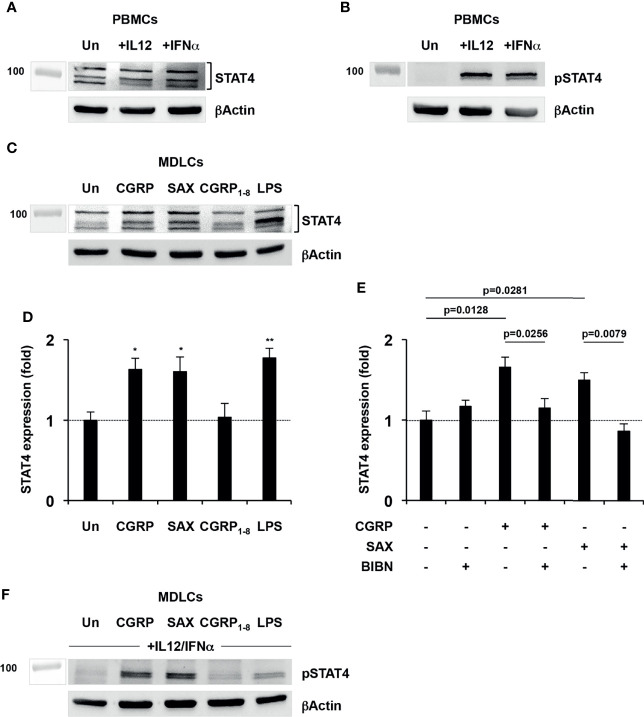
CGRP and SAX, but not CGRP_1–8_, increase STAT4 expression in MDLCs. **(A, B)** PHA/IL2-activated PBMCs were serum-starved overnight at 37°C, and left untreated (Un) or stimulated for 30 min with either IL12 or IFNα. Shown are representative Western blots (of n = 4 independent experiments using PBMCs from different individuals) of total STAT4 **(A)** and pSTAT4 **(B)** expression. **(C, D)** MDLCs were cytokine-starved overnight at 37°C, and treated with CGRP (0.1 μM), SAX (0.1 μM), CGRP_1–8_ (10 μM) or LPS (10 μg/ml) as positive control. In panel **(C)**, shown is a representative Western blot (of n = 4 independent experiments using MDLCs from different individuals) of total STAT4 expression. In panel **(D)**, shown are mean ± SEM folds expression of total STAT4, normalized to that of beta actin. *p < 0.0500, **p < 0.0050, two-sided Student’s t-test. **(E)** MDLCs were cytokine-starved overnight at 37°C and treated with CGRP (0.1 μM) or SAX (0.1 μM). The CGRP receptor antagonist BIBN4096 (BIBN, 1 μM) was added 15 min before addition of agonists. Shown are mean ± SEM (of n = 4 independent experiments using MDLCs from different individuals) folds expression of total STAT4, normalized to that of beta actin. **(F)** MDLCs were treated as described in panels **(C, D)** above, and further stimulated for 30 min with combination of IL12 + IFNα. Shown is a representative Western blot (of n = 4 independent experiments using MDLCs from different individuals) of pSTAT4 expression.

We previously discovered that CGRP inhibits MDLCs-mediated HIV-1 trans-infection *via* STAT4 (14), and therefore performed WB experiments to quantify STAT4 levels directly. Using activated PBMCs as positive control, we first confirmed the suitability of our Abs for detection of total STAT4 following cell activation (**Figure 5A** showing PBMCs from one representative individual of n = 4 tested; **Supplementary Figure 1** showing PBMCs from two of the additional individuals), as well as pSTAT4 following cell activation and additional 30 min cytokine stimulation (**Figure 5B**), using IL12 and IFNα that induce STAT4 phosphorylation (25). Next, we measured total STAT4 in MDLCs treated with CGRP, SAX or CGRP_1–8_, as well as with LPS as positive control [i.e., LPS increases STAT4 in dendritic cells (26)]. These experiments showed that like LPS, both CGRP and SAX significantly increased total STAT4 expression (**Figures 5C, D**). In contrast, the CGRP N-terminal fragment CGRP_1–8_ failed to increase STAT4 expression, which remained comparable to that in untreated MDLCs (**Figures 5C, D**). As for HIV-1 trans-infection and langerin expression described above, the CGRP receptor antagonist BIBN4096 abrogated CGRP- and SAX-mediated increase in STAT4 (**Figure 5E**). Finally, we confirmed that CGRP- and SAX-induced increased STAT4 was functional, as it could be readily phosphorylated upon subsequent cytokine stimulation with IL12 + IFNα (**Figure 5F**).

These results show that CGRP and SAX, but not CGRP_1–8_, increase expression of STAT4 that is implicated in inhibition of HIV-1 trans-infection in MDLCs.

### CGRP and SAX Inhibit Mucosal HIV-1 Transmission in Human Mucosal Tissues *Ex-Vivo*

We further tested the anti-HIV-1 activities of CGRP and SAX using our previously described models of human penile and inner foreskin tissue explants (11, 21). In these models, small pieces of human mucosal tissues are placed in two-chamber transwell inserts, and hollow cloning ring cylinders are adhered to their apical side using surgical glue, permitting for subsequent polarized exposure to HIV-1 that mimics viral transmission *in-vivo*. Of note, HIV-1 entry in these mucosal sites is induced by polarized exposure to HIV-1-infected cells, which form viral synapses with apical epithelial cells that lead to polarized HIV-1 budding. In contrast, cell-free HIV-1 inefficiently enters these epithelia (11, 21).

**Figure 6:**
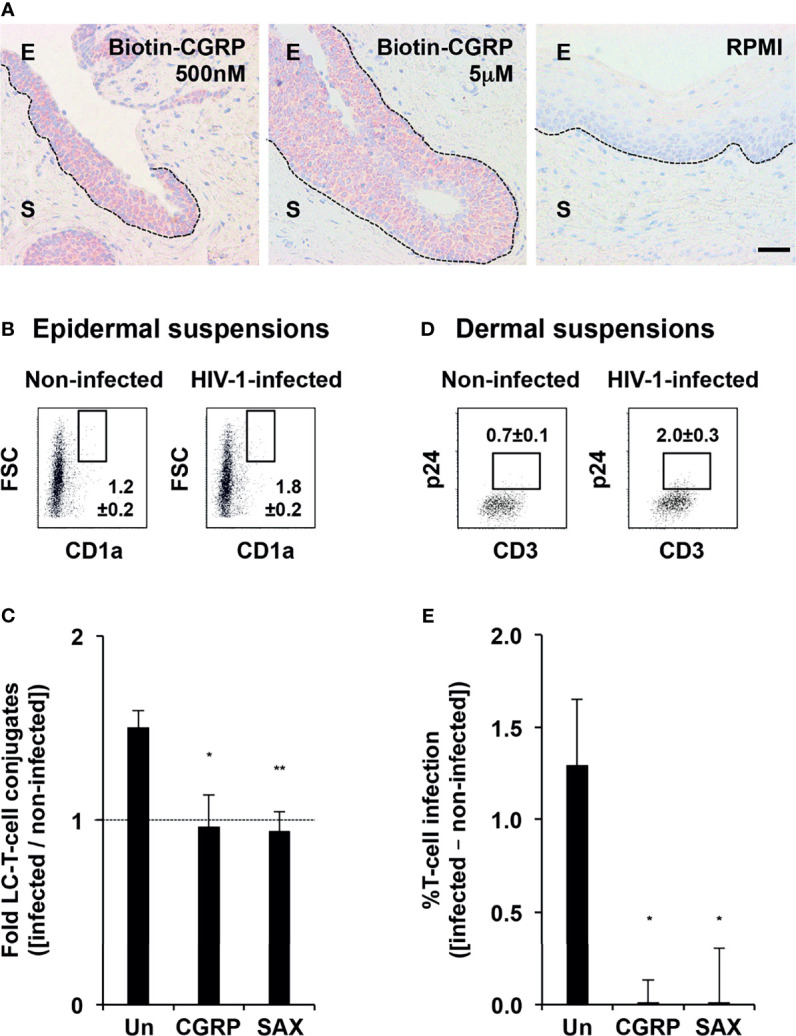
CGRP and SAX inhibit mucosal HIV-1 transmission in human mucosal tissues *ex-vivo*. **(A)** Entry of biotinylated CGRP into the epithelium of penile *fossa navicularis* explants, revealed with streptavidin-HRP, AEC peroxidase substrate (red), and hematoxyline counterstaining (blue). Images are representative of n = 3 tissues; broken lines denote the basement membranes and scale bar = 20 μm; E, epithelium and S, stroma. **(B, C)** Inner foreskin tissue explants were left untreated or pre-treated with CGRP or SAX (1 μM) for 24 h at 37°C. Explants were next inoculated in a polarized manner with either non-infected or HIV-1-infected PBMCs for 4 h, and immediately digested with dispase/trypsin. In panel **(B)**, shown are representative flow cytometry dot plots of epidermal cell suspensions triple stained for surface expression of CD3, CD8, and CD1a and examined by flow cytometry. Cells were gated on CD3+CD8- T-cells, and numbers represent mean ± SEM (of n = 4 independent experiments using tissues from different individuals) percentages of FSC^high^CD1a^high^ conjugates following inoculation with either non-infected or HIV-1-infected PBMCs. In panel **(C)**, graph shows mean ± SEM folds increase in conjugate percentages, calculated as [(% conjugates following inoculation with HIV-1-infected PBMCs)/(% conjugates following inoculation with non-infected PBMCs)]. *p = 0.0252 and **p = 0.0071 for CGRP or SAX vs. untreated, two-sided Student’s t-test. **(D, E)** Other explants were further incubated for additional three days and digested with collagenase/DNase. In panel **(D)**, shown are representative flow cytometry dot plots of dermal cell suspensions double stained for surface CD3 and intracellular p24 and examined by flow cytometry. Cells were gated on FSC^low^SSC^low^ lymphocytes, and numbers represent mean ± SEM (n = 4) percentages of CD3+p24+ cells following inoculation with either non-infected or HIV-1-infected PBMCs. In panel **(E)**, graph shows mean ± SEM percentages of HIV-1-infected T-cells, calculated as [(%CD3+p24+ cells following inoculation with HIV-1-infected PBMCs) − (%CD3+p24+ cells following inoculation with non-infected PBMCs)]; *p = 0.0249 and 0.0209 for CGRP or SAX vs. untreated, two-sided Student’s t-test.

To test for CGRP mucosal penetration, we prepared tissue explants from the stratified and non-keratinized penile *fossa navicularis* region that structurally resembles the vaginal epithelium, and added biotinylated CGRP to the apical side for 3 h, followed by histochemistry. These experiments showed that CGRP readily penetrated the epithelium, but not the stroma (**Figure 6A**).

We next pre-treated inner foreskin tissue explants for 24 h with CGRP or SAX at 1 μM, i.e., their molar concentration inducing maximal responses at similar efficiencies *in-vitro* (see **Figures 3A** and **4A**). Explants were next inoculated in a polarized manner with non-infected or HIV-1-infected PBMCs for 4 h. Epidermal cell suspensions were then immediately prepared and the percentages of high forward scatter (FSC) conjugates between LCs and T-cells were determined by flow cytometry. Of note, we focused on CD1a^high^ cells that represent the LC1 population, as CD1a^+^ cells in the inner foreskin include both the LC2 population and epidermal dendritic cells (27–29). In agreement with our previous results (11, 12), polarized exposure to HIV-1-infected PBMCs increased the percentages of FSC^high^CD1a^high^CD3^+^CD8^-^ conjugates (**Figure 6B**). As before, CGRP pre-treatment completely abrogated this increase (13), and a similar complete inhibitory effect was observed following SAX pre-treatment (**Figure 6C**).

In other experiments, dermal cell suspensions were prepared following additional incubation of explants in fresh medium for three days, and the percentages of HIV-1-infected T-cells were determined by flow cytometry. These experiments confirmed that polarized exposure to HIV-1-infected PBMCs resulted in HIV-1 infection of a small proportion of T-cells in the dermis (**Figure 6D**), and that CGRP completely blocked such infection (**Figure 6E**), as we reported (13). Similarly, SAX pre-treatment resulted in undetectable levels of HIV-1 p24^+^CD3^+^ dermal T-cells (**Figure 6E**).

These results indicate that both CGRP and SAX are highly effective in preventing mucosal HIV-1 transmission and infection within human mucosal tissues *ex-vivo*.

## Discussion

**Figure 7:**
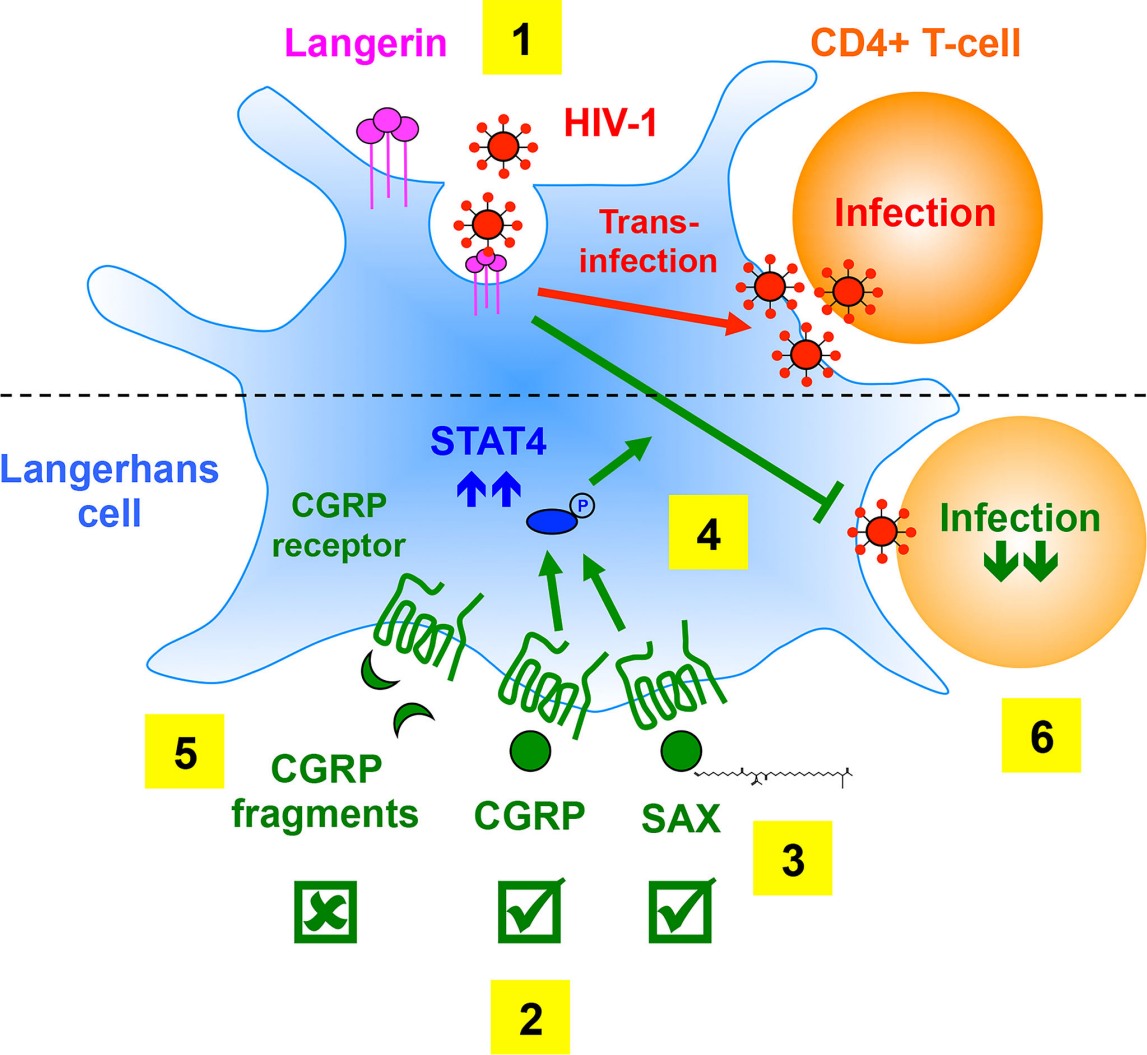
Summary of the requirements of CGRP receptor activation for inhibition of mucosal HIV-1 transmission. **(1)** In LCs, HIV-1 binding to langerin induces viral internalization and subsequent degradation, while virions escaping degradation trans-infect CD4+ T-cells. **(2)** We previously showed that CGRP activates its cognate receptor expressed by LCs and affects a multitude of cellular and molecular process (not shown), resulting in significant inhibition of mucosal HIV-1 trans-infection *in-vitro* and *ex-vivo*. **(3)** We show in the present study that SAX, a long-acting metabolically stable analogue of CGRP, also activates the CGRP receptor. **(4)** Both CGRP and SAX increase expression of langerin (not shown) and STAT4 (that can be readily phosphorylated upon subsequent cytokine stimulation), which result in inhibition of HIV-1 trans-infection *in-vitro* and *ex-vivo*. **(5)** In contrast, several CGRP peptide fragments fail to activate the CGRP receptor and to increase langerin/STAT4 expression, and accordingly lack anti-HIV-1 activity. **(6)** CGRP-mediated inhibition of HIV-1 dissemination from LCs to CD4+ T-cells might permit their long-term maintenance in the BLT model of mucosal HIV-1 infection *in-vivo*.

In the present study, we determined the requirements and utility of CGRP receptor activation, by CGRP receptor agonists, for the inhibition of mucosal HIV-1 transmission. These findings are schematically summarized in **Figure 7**.

Our results show that in order to block langerin-mediated HIV-1 trans-infection in MDLCs, CGRP receptor activation requires full-length CGRP or SAX, in contrast to CGRP peptide fragments that are ineffective. While SAX has >10-fold lower potency than CGRP, both agonists are similarly effective at their highest micromolar concentrations tested. We speculate that CGRP peptide fragments, previously reported to be functional (18, 19, 24), are ineffective in our experimental settings due to potential ‘biased signaling’ (30) across the CGRP receptor, which similar to other G-protein coupled receptors, activates multiple downstream signaling pathways (31). Hence, compared to the full-length native CGRP ligand and the analogue SAX, CGRP fragments might have allosteric bias for preferential activation of particular signaling pathways, which are not the ones mediating inhibition of HIV-1 trans-infection. In support of this hypothesis, our results show that activation of the CGRP receptor by CGRP and SAX results in an increased expression of langerin and STAT4, which are involved in the inhibition of HIV-1 trans-infection (14). In contrast, CGRP_1–8_ fails to increase langerin and STAT4 expression, and accordingly lacks anti-HIV-1 inhibitory activity. We also speculate that our bivalent CGRP peptide fragments might require further optimization to be rendered functional. For instance, the N+C-terminal fragments could be re-designed to better fit into the CGRP receptor binding pockets with higher affinity, by using longer, different and/or more flexible spacer regions.

In the current study we used our previously described model of human inner foreskin tissue explants *ex-vivo*, which represents an early snapshot of mucosal HIV-1 entry (11, 12). In this model, polarized exposure to cell-associated HIV-1 increases the formation of LC-T-cell conjugates in the epithelium (11, 12). Our results show that CGRP penetration is restricted to the epithelium, and that both CGRP and SAX completely inhibit the increase in LC-T-cell conjugates formation mediated by cell-associated HIV-1. Importantly, although CD4+ T-cells express the CGRP receptor and are CGRP-responsive (32, 33), we previously showed that CGRP treatment of CD4+ T-cells has no effect on HIV-1 trans-infection (13). We therefore speculate that CGRP- and SAX-mediated inhibition of CD4+ T-cells infection with HIV-1, which we further observed *ex-vivo*, is mediated by CGRP and SAX acting on inner foreskin LCs and reducing their capacity to disseminate HIV-1 to CD4+ T-cells across cellular conjugates.

We also tested the effects of CGRP *in-vivo* in normal and humanized BLT mice. The latter represents a complimentary model to our *ex-vivo* tissue explants, as it permits to follow mucosal HIV-1 transmission over time. By combining the results obtained in these different models, we propose potential explanations for CGRP-mediated long-term maintenance of CD4+ T-cells *in-vivo*. Hence, CGRP transiently controls the increase in viral loads and could inhibit the previously reported process of HIV-1 dissemination from vaginal LCs to CD4+ T-cells (34). In turn, these effects would result in limited HIV-1 infection and elimination of CD4+ T-cells, permitting their long-term maintenance in the BLT model. In contrast, CGRP-mediated effects are not mediated *via* T-cells recruitment (i.e., as we observed in normal mice), or direct inhibition of CD4+ T-cells infection with HIV-1 [that is one of the reported mechanisms mediating HIV-1 transmission in the vagina (34). Interestingly, both CGRP and SAX completely block HIV-1 transmission in inner foreskin tissue explants *ex-vivo*, but CGRP exerts only partial protection in BLT mice *in-vivo*. Such differences might be related to the duration of agonist pre-treatment, i.e., 24 h in tissue explants vs. 6 h in BLT mice.

Pre-exposure prophylaxis (PrEP) is currently available and is highly effective for the prevention of HIV-1 transmission. Yet, important barriers still limit PrEP efficacy and usage, such as adherence, cost, access, stigma, adverse side effects, and drug resistance (35). Therefore, alternative approaches are being developed to increase the range of biomedical HIV-1 prevention options, such as long-acting injectable formulations, broadly neutralizing Abs, vaginal rings, implants, dermal patches, and topical microbicides (36).

Collectively, our results provide proof-of-concept that CGRP receptor agonists are useful in blocking HIV-1 transmission in complex mucosal settings. We suggest that in order to achieve better and long-lasting viremia and CD4+ T-cell control, treatment with CGRP receptor agonists should be longer, with repeated and continuous applications. In parallel, novel HIV-1 prophylactic formulations/devices could be developed, which would permit a slow release of optimized agonists of CGRP and/or higher potency mucosal metabolically stable derivatives. As such, HIV-1 infection should be included within the different pathologies and inflammatory conditions, in which CGRP is beneficial and could be harnessed to exert protective clinical effects.

## Data Availability Statement

The original contributions presented in the study are included in the article/**Supplementary Material**. Further inquiries can be directed to the corresponding authors.

## Ethics Statement

The studies involving human participants were reviewed and approved by the Comités de Protection des Personnes (CPP Paris-IdF XI, N.11016). The patients/participants provided their written informed consent to participate in this study. The animal study was reviewed and approved by the institutional review board of the San Raffaele Scientific Institute (IACUC no. 599).

## Author Contributions

YG and MB conceived the study and designed the experiments. YG, JM, EC, and ASe performed the experiments. ASa provided SAX and its experimental requirements. GS, FS, and LL designed and performed experiments using normal mice. ND and MZ provided foreskin tissues. YG wrote the paper. All authors made a substantial, direct and intellectual contribution to the work, and approved it for publication.

## Funding

The study was funded by research grants from the La SATT IDFinnov (maturation project n°005 to YG and MB), the Agence Nationale de la Recherches sur le Sida et les Hépatites virales (ANRS) | Maladies Infectieuses Émergentes (ECTZ159208 to YG), the Fondation pour la Recherche Medicale (FRM EQU201903007830; to MB). The funder was not involved in the study design, collection, analysis, interpretation of data, the writing of this article or the decision to submit it for publication. JM, EC and ASe were supported by fellowships from the ANRS.

## Conflict of Interest

The authors declare that the research was conducted in the absence of any commercial or financial relationships that could be construed as a potential conflict of interest.

## Publisher’s Note

All claims expressed in this article are solely those of the authors and do not necessarily represent those of their affiliated organizations, or those of the publisher, the editors and the reviewers. Any product that may be evaluated in this article, or claim that may be made by its manufacturer, is not guaranteed or endorsed by the publisher.
