# Supplementary material for: Native CGRP Neuropeptide and Its Stable Analogue SAX, But Not CGRP Peptide Fragments, Inhibit Mucosal HIV-1 Transmission
Source: Front Immunol. 2021 Dec 8;12:785072. doi: 10.3389/fimmu.2021.785072 (PMC8692891; doi:10.3389/fimmu.2021.785072)
Supplement: Supplementary file 1 [file DataSheet_1.pdf]

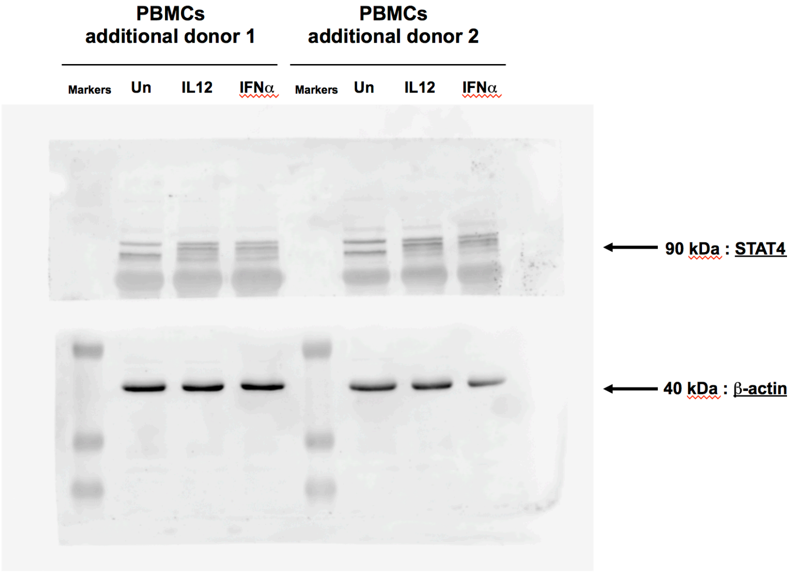

Supplementary Figure 1. Detection of STAT4 in PBMCs by WB. PHA/IL2-activated PBMCs, prepared from two additional individuals, were serum-starved overnight at 37°C, and left untreated (Un) or stimulated for 30min with either IL12 or IFNα. WB was next used to determine expression of total STAT4 and beta actin, as described in the Methods section. Shown is the whole blot, which was cut in two and incubated with either rabbit polyclonal anti-human STAT4 Ab (Proteintech #13028-1AP, 0.5mg/ml; upper part of blot) or beta actin (bottom part of blot).
